# Genome Sequencing as a Diagnostic Test in Children With Unexplained Medical Complexity

**DOI:** 10.1001/jamanetworkopen.2020.18109

**Published:** 2020-09-22

**Authors:** Gregory Costain, Susan Walker, Maria Marano, Danielle Veenma, Meaghan Snell, Meredith Curtis, Stephanie Luca, Jason Buera, Danielle Arje, Miriam S. Reuter, Bhooma Thiruvahindrapuram, Brett Trost, Wilson W. L. Sung, Ryan K. C. Yuen, David Chitayat, Roberto Mendoza-Londono, D. James Stavropoulos, Stephen W. Scherer, Christian R. Marshall, Ronald D. Cohn, Eyal Cohen, Julia Orkin, M. Stephen Meyn, Robin Z. Hayeems

**Affiliations:** 1 Division of Clinical and Metabolic Genetics, The Hospital for Sick Children, Toronto, Ontario, Canada; 2 Centre for Genetic Medicine, The Hospital for Sick Children, Toronto, Ontario, Canada; 3 The Centre for Applied Genomics, The Hospital for Sick Children, Toronto, Ontario, Canada; 4 Genetics and Genome Biology, Research Institute, The Hospital for Sick Children, Toronto, Ontario, Canada; 5 Division of Paediatric Medicine, The Hospital for Sick Children, Toronto, Ontario, Canada; 6 Child Health Evaluative Sciences, Research Institute, The Hospital for Sick Children, Toronto, Ontario, Canada; 7 Department of Molecular Genetics, University of Toronto, Toronto, Ontario, Canada; 8 The Prenatal Diagnosis and Medical Genetics Program, Mount Sinai Hospital, Toronto, Ontario, Canada; 9 Department of Paediatrics, University of Toronto, Toronto, Ontario, Canada; 10 Genome Diagnostics, Department of Paediatric Laboratory Medicine, The Hospital for Sick Children, Toronto, Ontario, Canada; 11 Laboratory Medicine and Pathobiology, University of Toronto, Toronto, Ontario, Canada; 12 Department of Paediatrics, University of Toronto, Toronto, Ontario, Canada; 13 Institute of Health Policy Management and Evaluation, University of Toronto, Toronto, Ontario, Canada; 14 Center for Human Genomics and Precision Medicine, University of Wisconsin, Madison

## Abstract

**Importance:**

Children with medical complexity (CMC) represent a growing population in the pediatric health care system, with high resource use and associated health care costs. A genetic diagnosis can inform prognosis, anticipatory care, management, and reproductive planning. Conventional genetic testing strategies for CMC are often costly, time consuming, and ultimately unsuccessful.

**Objective:**

To evaluate the analytical and clinical validity of genome sequencing as a comprehensive diagnostic genetic test for CMC.

**Design, Setting, and Participants:**

In this cohort study of the prospective use of genome sequencing and comparison with standard-of-care genetic testing, CMC were recruited from May 1, 2017, to November 30, 2018, from a structured complex care program based at a tertiary care pediatric hospital in Toronto, Canada. Recruited CMC had at least 1 chronic condition, technology dependence (child is dependent at least part of each day on mechanical ventilators, and/or child requires prolonged intravenous administration of nutritional substances or drugs, and/or child is expected to have prolonged dependence on other device-based support), multiple subspecialist involvement, and substantial health care use. Review of the care plans for 545 CMC identified 143 suspected of having an undiagnosed genetic condition. Fifty-four families met inclusion criteria and were interested in participating, and 49 completed the study. Probands, similarly affected siblings, and biological parents were eligible for genome sequencing.

**Exposures:**

Genome sequencing was performed using blood-derived DNA from probands and family members using established methods and a bioinformatics pipeline for clinical genome annotation.

**Main Outcomes and Measures:**

The primary study outcome was the diagnostic yield of genome sequencing (proportion of CMC for whom the test result yielded a new diagnosis).

**Results:**

Genome sequencing was performed for 138 individuals from 49 families of CMC (29 male and 20 female probands; mean [SD] age, 7.0 [4.5] years). Genome sequencing detected all genomic variation previously identified by conventional genetic testing. A total of 15 probands (30.6%; 95% CI 19.5%-44.6%) received a new primary molecular genetic diagnosis after genome sequencing. Three individuals had novel diseases and an additional 9 had either ultrarare genetic conditions or rare genetic conditions with atypical features. At least 11 families received diagnostic information that had clinical management implications beyond genetic and reproductive counseling.

**Conclusions and Relevance:**

This study suggests that genome sequencing has high analytical and clinical validity and can result in new diagnoses in CMC even in the setting of extensive prior investigations. This clinical population may be enriched for ultrarare and novel genetic disorders. Genome sequencing is a potentially first-tier genetic test for CMC.

## Key Points

**Question:**

What is the diagnostic yield of genome sequencing in children with unexplained medical complexity and prior negative results of genetic testing?

**Findings:**

In this cohort study that included 138 individuals from 49 families, genome sequencing detected all genomic variation previously identified by conventional genetic testing and resulted in a new diagnosis for 31% of patients.

**Meaning:**

This study suggests that, because of its high yield, comprehensive nature, and increasingly competitive costs, genome sequencing is a potentially first-tier genetic test for children with unexplained medical complexity.

## Teaser

This cohort study evaluates the analytical and clinical validity of genome sequencing as a comprehensive diagnostic genetic test for children with medical complexity.

## Introduction

Children with medical complexity (CMC)^1,2,3,4^ have at least 1 chronic condition, technology dependence, multiple subspecialist involvement, and substantial health care use. Although these children compose less than 1% of the pediatric population, they account for 33% of all pediatric health care spending.^2^ A genetic cause is suspected in a large proportion of CMC, but most remain undiagnosed with conventional genetic testing.^5^ For many families, the diagnostic process is time intensive, resource intensive, and emotionally intensive.^5^ Children with medical complexity are a priority population for testing novel interventions.^1,6^ Genome sequencing has the potential to enhance the efficiency and effectiveness of diagnostic genetic testing in pediatric medicine,^7,8^ including in CMC.

Collectively, rare genetic conditions are an important cause of severe pediatric morbidity and mortality.^9,10^ A genetic diagnosis can inform prognosis, anticipatory care, management, and reproductive planning. Chromosomal microarray analysis (CMA)^7,11,12,13^ and exome sequencing (ES)^13,14,15,16,17^ are now established clinical genetic tests in resource-rich countries for a range of pediatric presentations. Genome sequencing offers several advantages compared with both CMA and ES^8,18^ and is a comprehensive genetic test potentially capable of detecting nearly all sequence and structural variation in the human genome.^7,8,17,19,20,21,22,23,24,25^ Rapid genome sequencing as a first-tier test in neonatal and pediatric intensive care units has been associated with a high diagnostic yield and potential health care cost savings.^22,23,24,25,26^ In contrast, genome sequencing is understudied in other settings.^7,8,20,27^

The goal of this observational cohort study in a population of CMC was to evaluate the analytical and clinical validity of genome sequencing as a genetic test.^28,29^ We anticipated that genome sequencing would be a high-yield and comprehensive testing strategy and that the undiagnosed CMC population may be enriched for rare and novel genetic disorders.

## Methods

### Recruitment, Inclusion and Exclusion Criteria, and Phenotyping

We recruited CMC younger than 18 years from a structured complex care program^30^ based at a tertiary care pediatric hospital during an 18-month period (May 1, 2017, to November 30, 2018). The standard operational definition for CMC has been published elsewhere.^31^ Families were eligible to participate if an underlying genetic condition was suspected in the child (proband) but had not been established by conventional genetic testing. The study size was limited by available funding to a maximum of 50 families. Children with genetic diagnoses that explained only a component of their primary phenotype and those with a variant of uncertain significance that could represent a diagnosis were included. Exclusion criteria were the following: the child was no longer actively followed up by the complex care program, neither biological parent was available for the study, genetic testing was in progress, and the child was involved in another research study of genome sequencing. All probands were seen in consultation by a clinical geneticist at the time of enrollment (if not already seen within the last 12 months) to ensure access to standard-of-care testing. Phenotype and family history data were extracted from the electronic medical record and entered into PhenoTips.^32^ Phenotypic information is represented in PhenoTips using the Human Phenotype Ontology.^33^ Data regarding conventional molecular genetic testing were also extracted from the electronic medical record. This study followed the Strengthening the Reporting of Observational Studies in Epidemiology (STROBE) guideline for observational cohort studies.^34^ The study was approved by the Research Ethics Board at The Hospital for Sick Children. Parents and/or guardians provided written consent on their child’s behalf. Where appropriate, children provided written and/or oral assent.

### Genome Sequencing and Variant Annotation

Genome sequencing was performed at the Centre for Applied Genomics (Toronto, Ontario, Canada) using established methods,^8^ with high-quality DNA extracted from whole blood. In brief, library preparation was performed from 500 ng of DNA using the TruSeq Nano DNA Library Preparation Kit (Illumina Inc) omitting the polymerase chain reaction amplification step, followed by sequencing on a HiSeq X platform (Illumina Inc) per recommended protocols. Base calling and data analysis were performed using Bcl2FASTQ or HiSeq Analysis Software version 2-2.5.55.1311 (Illumina Inc) and reads were mapped to the hg19 reference sequence using Burrows-Wheeler Aligner, version 0.7.12 (Illumina Inc). Single-nucleotide variations (SNVs) and indels were detected using Genome Analysis Toolkit, version 3.4-46 or version 3.7 (Broad Institute). Detected variants were annotated using a custom pipeline based on ANNOVAR (ANNOtate VARiation; Center for Applied Genomics, University of Pennsylvania)^35^ as previously described^8^ and with the addition of SpliceAI (Illumina Inc).^36^ Copy number variations (CNVs) were detected using the read depth methods ERDS (Estimation by Read Depth with Single-nucleotide variants; Duke University)^37^ and CNVnator (Yale University)^38^ with a window size of 500 bp. High-quality CNVs were defined as those detected by ERDS that were also detected by CNVnator with greater than 50% reciprocal overlap.^39^ Structural variants were detected using the algorithms Manta (Illumina Inc),^40^ LUMPY (University of Virginia),^41^ and DELLY (European Molecular Biology Laboratory).^42^ Structural variants that were detected by at least 2 callers were prioritized, with variants supported by at least 5 paired or split reads considered as higher stringency. Short tandem repeats were genotyped at 54 targeted loci of known or potential clinical relevance using ExpansionHunter version 3.1.2 (Illumina Inc).^43^ Mitochondrial variants were converted to NC_012920 coordinates with a custom script and then annotated using MitImpact19 version 2.4^44^ (Laboratory of Bioinformatics, IRCCS Casa Sollievo della Sofferenza). Where necessary, read alignments were manually inspected using Integrative Genomics Viewer^45^ (Broad Institute). Rare SNVs and indels were defined as those present at less than 1% allele frequency in large population control data sets,^46,47,48^ and rare structural variants and CNVs were those present in less than 1% of unaffected parents in the Autism Speaks MSSNG data set.^49^ Copy number variations were also annotated with respect to the degree of overlap with those in the Database of Genomic Variants.^50,51^ Genome sequencing data for this study will be deposited in the European Genome-Phenome Archive.

### Interpretation and Clinical Confirmation of Variants

Candidate variants that were deemed relevant to the primary phenotype according to established laboratory reporting criteria^52^ were discussed with the clinical team and designated as diagnostic by consensus. Diagnostic variants in established disease genes were classified as likely pathogenic or pathogenic using the American College of Medical Genetics and Genomics criteria.^52^ Maternity and paternity were confirmed for putative de novo variants. In 3 instances (CMC 21 and *THOC2* [OMIM 300395] variant, CMC 24 and *CLCN4* [OMIM 302910] variant, and CMC 38 and *CAD* [OMIM 114010]) variant), additional functional studies supportive of a damaging association with the gene or gene product were facilitated by international collaborators.^53,54,55^ We also reported secondary findings in American College of Medical Genetics and Genomics Secondary Findings version 2.0 genes^56^ with potential childhood-onset phenotypes. All diagnostic variants were confirmed by an orthogonal method in a laboratory with Clinical Laboratory Improvement Amendments and College of American Pathologists certification. Changes in medical management triggered by the genome sequencing results were recorded by the clinical team.

### Statistical Analysis

All analyses were performed using R statistical software, version 4.0.2 (R Foundation for Statistical Computing). Fisher exact test for comparison of proportions and Kruskal-Wallis test for comparison of medians were used for within-group comparisons. All *P* values were from 2-tailed tests and *P* < .05 was considered statistically significant.

## Results

**Figure 1.**
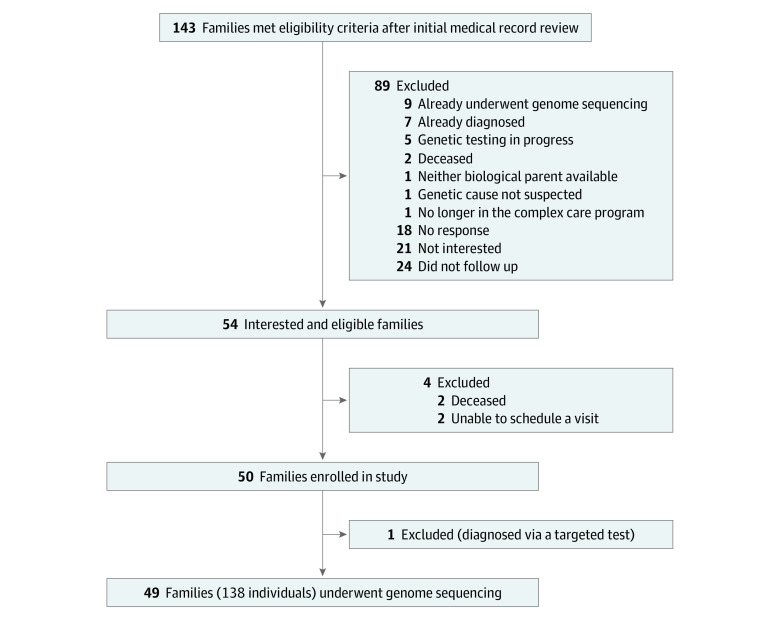
Study Recruitment Flowchart

Review of the care plans^30^ for 545 CMC identified 143 families who appeared to meet eligibility criteria (Figure 1). Fifty-four families met inclusion criteria and were interested in participating, of which 50 were assigned research identification numbers (Figure 1). Prior to genome sequencing, 1 proband (CMC 27) was found through detailed medical record review to have had a diagnostic variant detected in the course of another research study. The result was clinically confirmed and disclosed to the care team for the first time. This individual was excluded from the present study so as not to artificially inflate the diagnostic yield of genome sequencing, leaving 49 participating families.

### Phenotype and Family History Characteristics

Of the 49 probands who underwent genome sequencing, 29 (59.2%) were boys. The mean (SD) age was 7.0 (4.5) years. The self-reported races/ethnicities were European or White (n = 26), South Asian (n = 10), other or mixed (n = 5), Middle Eastern (n = 4), Ashkenazi Jewish (n = 3), and East Asian (n = 1). Six probands (12.2%) had a first-degree relative with at least partial phenotypic overlap, and there was parental consanguinity for 5 probands (10.2%). All probands met criteria for medical complexity as a consequence of congenital anomalies and/or neurologic or developmental features. The median number of Human Phenotype Ontology terms coded per proband was 24 (range, 6-58). The 1219 total features were distributed across 15 phenotypic categories (eFigure 1 in the Supplement), and each category was represented in 8 individuals or more (eFigure 2 in the Supplement). The most frequently represented category was neurologic or developmental, with 288 total features (23.6%; eFigure 1 in the Supplement) and at least 1 feature in this category in 47 probands (95.9%; eFigure 2 in the Supplement).

### Conventional Genetic Testing

The median number of conventional genetic tests per proband was 4 (range, 1-13), and a total of 232 tests were performed in this patient cohort (eFigure 3 in the Supplement). Six individuals met inclusion criteria but were nonetheless known at the time of recruitment to have variants that might explain at least part of their phenotype (eTable 1 in the Supplement). Testing organized at the time of enrollment in this study included 9 CMA tests, 7 ES tests, 2 next-generation sequencing gene panel tests, and 2 single-gene tests. By the completion of the study, 48 probands (98.0%) had undergone CMA testing and 33 (67.3%) had undergone ES (eFigure 4 in the Supplement). Of the 16 probands who did not undergo ES, 7 did not meet clinical eligibility criteria within the provincial health care system, 4 were diagnosed by genome sequencing before ES was approved for funding and initiated, 3 were offered ES but the families did not follow up, and 2 were diagnosed by next-generation sequencing gene panel tests.

### Genome Sequencing Coverage and Analytical Validity

We performed genome sequencing for 138 individuals from 49 families. This included 40 parent-child trios, 4 singletons (child only; these were not upgraded to trios once parent samples became available because diagnoses had already been made), 3 mother-child pairs (the fathers were unavailable), and 2 quartets (parent-child trio with affected sibling). Across the cohort, the mean depth of coverage of genome sequencing was 36X (eFigure 5 in the Supplement). The median percentage of base pairs with genome-wide coverage at least 10X was 97% and at least 20X was 95%.

In total across the study cohort, 132 genomic variants were identified by clinical genetic testing and reported back to the ordering clinician (106 SNVs or indels, 17 CNVs, 7 short tandem repeat lengths, and 2 mitochondrial DNA variants). These were mostly categorized as either variants of uncertain significance or likely benign. For 8 putative variants across 5 individuals that were not detected by genome sequencing, the clinical result was later retracted or discounted. For 3 variants this was because of sample mix-ups; the remaining 5 SNVs failed Sanger confirmation and/or were also not detected by an orthogonal clinical test (eg, clinical ES). Genome sequencing detected the remaining 124 variants (100%), indicating excellent analytical validity.

### Primary Diagnostic Findings From Genome Sequencing

**Figure 2.**
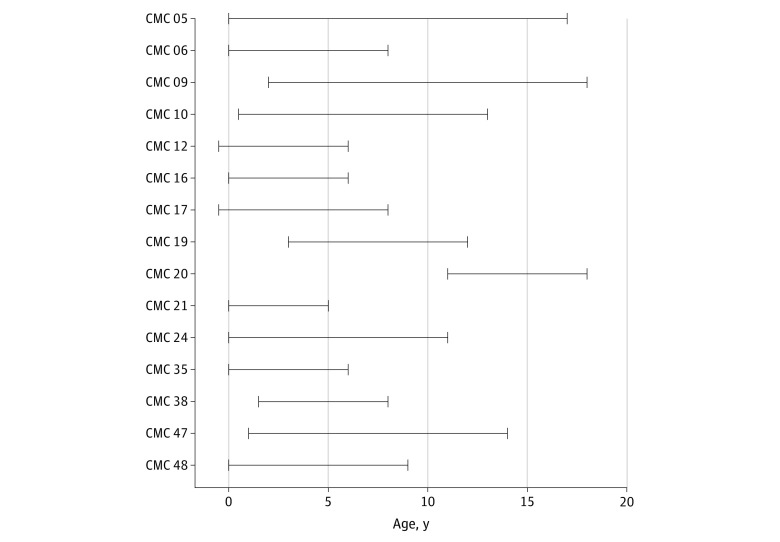
Timeline of the Diagnostic Process Horizontal lines indicate the duration of the diagnostic process from initial suspicion for an underlying genetic condition to diagnosis by genome sequencing.

In total, 15 of 49 probands (30.6%; 95% CI, 19.5%-44.6%) received a new primary molecular genetic diagnosis by genome sequencing during the study period (Table 1).^53,57,58,59,60,61,62,63^ There were no marked differences in demographic or clinical features between the diagnosed and undiagnosed subgroups, aside from a higher median age in the diagnosed subgroup (eTable 2 and eFigure 6 in the Supplement). Concerns for an underlying genetic condition were first documented prenatally or in the immediate neonatal period in 10 of the 15 probands (66.7%), and the median duration of the diagnostic process (from first clinical genetic test to disclosure of diagnosis) was 8 years (range, 5-17 years) (Figure 2). Most diagnostic variants were exonic sequence-level variants (Table 1).^53,57,58,59,60,61,62,63^ A maternally inherited single-exon duplication in the X chromosome gene *KDM6A* (OMIM 300128) causing Kabuki syndrome in CMC 16 was not detected by CMA, ES, or an initial multiplex ligation-dependent probe amplification test of that gene.

**Table 1.** 16 Primary Diagnostic Variants Identified by Genome Sequencing in 15 Study Participants

Study ID	Sex	Selected features	Gene	MIM No. gene (phenotype)	IP	Variant details (zygosity) [transcript]	Origin	Associated human phenotype^a^
CMC 05	M	GDD or ID, CNS anomalies	*RAC3*	602050 (NA)	AD	c.182A>T / p.(Gln61Leu) (het) [NM_005052.2]^b^	De novo	Novel disorder^57^
CMC 06	M	GDD or ID, MCA, craniofacial, other^c^	*HDAC8*	300269 (300882)	XL	c.134_137del / p.(Ile45Lysfs*9) (hem) [NM_018486.2]^b^	De novo	Rare disorder
CMC 09	M	GDD or ID, seizures, cerebral atrophy	*H3F3B*	601058 (NA)	AD	c.365C>G / p.(Pro122Arg) (het) [NM_005324.4]	De novo	Novel disorder
CMC 10	F	GDD or ID, microcephaly	*CASK*	300172 (300749)	XL	c.1685dup / p.(Ser562Argfs*18) (het) [NM_003688.3]	De novo	Ultrarare disorder
CMC 12	F	GDD or ID, seizures, HL, CNS anomalies	*PDHA1*	300502 (312170)	XL	c.937_940dup / p.(Ser314Lysfs*3) (het) [NM_000284.3]^b^	De novo	Rare disorder
CMC 16	M	GDD or ID, craniofacial, other^c^	*KDM6A*	300128 (300867)	XL	chrX:44818001-44826000×2	Maternal	Rare disorder
CMC 17	F	GDD or ID, seizures, constipation	*FBXW7*	606278 (NA)	AD	c.1920C>A / p.(Ser640Arg) (het) [NM_033632.3]	De novo	Novel disorder
CMC 19	F	GDD or ID, seizures, ASD	*STXBP1*	602926 (612164)	AD	c.1454T>A / p.(Ile485Asn) (het) [NM_003165.3]^b^^,^^d^	De novo	Rare disorder
CMC 20	F	GDD or ID, CNS anomalies	*NKX6-2*	605955 (617560)	AR	c.234del / p.(Leu79Cysfs*109) (hom) [NM_177400.2]^a^	Maternal and paternal	Ultrarare disorder
CMC 21	M	GDD, seizures, respiratory	*THOC2*	300395 (300957)	XL	c.229C>T / p.(Arg77Cys) (hem) [NM_001081550.1]^b^	De novo	Ultrarare disorder
CMC 24	M	GDD or ID, seizures, ASD, other^c^	*CLCN4*	302910 (300114)	XL	c.1106C>T / p.(Pro369Leu) (hem) [NM_001830.3]	De novo	Rare disorder
CMC 35	F	GDD, macrocephaly, CNS anomalies	*PIK3CA*	171834 (602501)	AD	c.1093G>A / p.(Glu365Lys) (het) [NM_006218.2]^b^	De novo	Rare disorder
CMC 38	F	GDD or ID, regression, seizures, anemia	*CAD*	114010 (616457)	AR	c.1576G>A / p.(Gly526Arg) (hom) [NM_004341.4]	Maternal and paternal	Ultrarare disorder
CMC 47	M	GDD or ID, seizures, CNS anomalies, other^c^	*FOXG1*	164874 (613454)	AD	c.177_186del / p.(Pro60Argfs*129) (het) [NM_005249.3]	De novo	Rare disorder
CMC 48	F	Microphthalmia, sclerocornea, Peters anomaly, aphakia^e^	*PXDN*	605158 (269400)	AR	c.1569_1570insT / p.(Thr524Tyrfs*53) (het)	Maternal	Ultrarare disorder
						c.3206C>A / p.(Ala1069Asp) (het)	Paternal	
						[NM_012293.2]		

Abbreviations: AD, autosomal dominant; AR, autosomal recessive; ASD, autism spectrum disorder; CNS, central nervous system; F, female; GDD, global developmental delay; hem, hemizygous; het, heterozygous; HL, hearing loss; hom, homozygous; ID, intellectual disability; IP, inheritance pattern; M, male; MCA, multiple congenital anomalies; MIM, Mendelian Inheritance in Man; NA, not available; XL, X chromosome–linked.

^a^ Ultrarare was defined as there being fewer than approximately 25 reported individuals in the scientific literature (as of August 2019). We used what is likely a more conservative definition of ultrarare than the European Parliament (“diseases affecting no more than one person in 50 000”)^58^ because of inadequate population incidence and prevalence data.

^b^ ClinVar Accession Number: VCV000585005.1 (*RAC3*; same patient); VCV000211139.1 (*HDAC8*; different patient); VCV000214945.1 (*PDHA1*; same patient); VCV000595655.1 (*STXBP1*; different patient); VCV000504099.2 (*NKX6-2*; same patient); VCV000488436.1 (*THOC2*; different patient); VCV000419222.3 (*PIK3CA*; different patient).

^c^ Atypical but previously reported feature seen in association with the genetic diagnosis: choanal stenosis or atresia and intermittent cytopenias (CMC 06) each in at least 1 individual with Cornelia de Lange syndrome^59,60,61^; hyperinsulinemic hypoglycemia (CMC 16) may be an underappreciated feature of Kabuki syndrome^62^; congenital diaphragmatic hernia (CMC 24) in at least 1 individual with *CLCN4*-related disorder^53^; and congenital microcephaly (CMC 47) in at least 1 individual with *FOXG1*-related disorder.^63^

^d^ Mosaic variant in blood.

^e^ Additional features in this participant that are not explained by the *PXDN* variants (and also are absent in her monozygotic twin) include intrauterine growth retardation, seizures, unilateral renal dysplasia, and hemihypertrophy of lower limb.

These study participants contributed to the discovery of 3 new genetic conditions (Table 1),^53,57,58,59,60,61,62,63^ including *RAC3* (OMIM 602050)-related disorder^57^ and 2 novel autosomal dominant neurodevelopmental syndromes that have been delineated through international collaborations.^64,65^ By conservative measures, another 9 probands had either ultrarare genetic conditions (fewer than approximately 25 reported individuals in the scientific literature) or very rare genetic conditions with 1 or more atypical features (Table 1).^53,57,58,59,60,61,62,63^ Selected variants of uncertain significance as well as selected variants in genes not yet associated with a human phenotype are reported in eTable 3 in the Supplement. A small deletion of uncertain significance overlapping *TLK2* (OMIM 608439) and 3 deep intronic variants of uncertain significance were not detected by ES (eTable 3 in the Supplement). In particular, the biallelic variants in *JAM3* (OMIM 606871; NM_032801) are suspected to be diagnostic despite c.256 + 1260G>C classifying as a variant of uncertain significance (eTable 3 in the Supplement). Entries were made in GeneMatcher^66^ for each gene of unknown significance. In addition to the 15 probands who received genetic diagnoses with genome sequencing, 1 proband (CMC 23) received a clinical diagnosis of PHACE (posterior fossa malformations, hemangioma, arterial anomalies, cardiac defects, eye anomalies) syndrome^67^ at the time of enrollment after excluding the phenotypic features explained by a pathogenic *HNF4A* (OMIM 616026) variant.

### Clinical Implications of Diagnostic Information

All primary diagnostic variants informed genetic and reproductive counseling. Four diagnoses had a sibling recurrence risk of 25% or more, and the remainder were the result of apparent de novo variants with a low (≤1%) empirical recurrence risk (Table 1).^53,57,58,59,60,61,62,63^ There were also reportable secondary findings in 2 probands (pathogenic variants in *MYH7* [OMIM 160760] and *LDLR* [OMIM 606945], respectively; Table 2^68,69^), which were inherited from previously undiagnosed parents. In total, 7 of the 49 families who participated in this study received diagnoses (via primary diagnostic variants, secondary findings, or new clinical diagnoses) that had immediate implications for medical management (Table 2).^68,69^ Targeted therapy was initiated for a child with uridine-responsive epileptic encephalopathy.^70^ At least 5 other diagnoses had published guidelines with management and surveillance recommendations^68^ and/or specific interventions listed in the Clinical Genomic Database.^71^

**Table 2.** Management Implications Beyond Reproductive Risk Counseling Resulting From Study Diagnoses

Study ID	Condition	Selected management implications
**Immediate implications for medical management**		
CMC 06	Cornelia de Lange syndrome	Clinical practice guidelines and syndrome-specific growth curves
CMC 16	Kabuki syndrome	Clinical practice guidelines and syndrome-specific growth curves
CMC 17	*MYH7*-related cardiomyopathy^a^	Echocardiogram and electrocardiogram, surveillance, and cascade testing in family
CMC 20	Familial hypercholesterolemia^b^	Lipid profiling and surveillance and cascade testing in family
CMC 23	PHACE syndrome^c^	Magnetic resonance angiography of brain, neck, and aortic arch
CMC 35	*PIK3CA*-related overgrowth syndrome	Screening for overgrowth-associated malignant neoplasm^d^
CMC 38	Uridine-responsive epileptic encephalopathy	Uridine supplementation
**General recommendations only**		
CMC 10	*CASK*-related disorder	Published guidelines with management and surveillance recommendations,^68^ and specific intervention listed in CDG (regarding risk of hearing impairment)
CMC 12	Pyruvate dehydrogenase complex deficiency	Published guidelines with management and surveillance recommendations,^68^ and specific intervention listed in CDG (regarding possible dietary and medical therapy)
CMC 19	*STXBP1* encephalopathy with epilepsy	Published guidelines with management and surveillance recommendations^68^
CMC 20	*NKX6-2*–related disorder	Published guidelines with management and surveillance recommendations^68^
CMC 48	Anterior segment dysgenesis 7	Specific intervention listed in CDG (regarding risk of glaucoma)

Abbreviations: CDG, Clinical Genomic Database; PHACE, posterior fossa malformations, hemangioma, arterial anomalies, cardiac defects, eye anomalies.

^a^ Likely pathogenic *MYH7* variant (NM_000257:c. 3158G>A): heterozygous and inherited from father.

^b^ Pathogenic *LDLR* variant (NM_000527:c.1476_1477del): heterozygous and inherited from mother.

^c^ Clinical diagnosis (see text for details).

^d^ Targeted therapy is also in development.^69^

## Discussion

More than 20% of 545 children in a clinically heterogeneous and well-phenotyped population of undiagnosed CMC were suspected to have a genetic disorder that had not yet been diagnosed by conventional genetic testing. In a subset of 49 families who underwent genome sequencing, the diagnostic yield was 30.6%. Several diagnoses had clinical implications that extended beyond genetic and reproductive counseling. The phenotypic complexity and extensive prior genetic testing with negative results were likely associated with the apparent enrichment, compared with other pediatric populations that undergo ES, for novel, ultrarare, and atypical presentations of rare genetic conditions. Genome sequencing detected all genetic variation identified by prior tests. These findings support a role for genome sequencing as a first-tier genetic test in CMC, and more generally as a cornerstone for use in pediatric undiagnosed disease programs.^72^

Establishing definitive genetic diagnoses for CMC can enable a better understanding of disease progression, guide medical care, and inform reproductive planning. Nonetheless, the importance of obtaining a genetic diagnosis may be underappreciated by some traditional metrics.^73^ Many parents reported the value of receiving positive results even in the absence of specific anticipatory care or management recommendations. This finding aligns with related literature that reflects on the intrinsic value of a diagnosis.^74,75^ The benefits associated with diagnosis are not restricted to young children and their parents. Rereferral for clinical genetics assessment should be considered for older children and teenagers with unexplained medical complexity who have not undergone genome-wide sequencing. Additional recommendations to improve the integration of genomics into the care of CMC include representation in care maps and care plans,^30,76^ review of prior clinical genetic testing results at each visit, inclusion of a genetics health care professional in multidisciplinary case review, and periodic consideration of the role for further genetic testing (for fully or partially undiagnosed patients) or of the potential implications for management and opportunities to participate in rare disease research (for diagnosed patients). Trio genome-wide sequencing is associated with a higher diagnostic yield than only the proband undergoing sequencing,^23^ and in our study facilitated novel disease gene discovery. However, 1 or both biological parents being unavailable for testing is not an absolute contraindication to clinical genome-wide sequencing.

The potential value of a genome sequencing result that shows no primary genetic diagnostic findings has not been clearly established. Reannotation and reanalysis of existing genome sequencing data can result in new diagnoses even after a relatively brief period of time.^19^ However, in specific clinical contexts, a lack of any diagnostic or candidate variants reduces the likelihood of a typical mendelian disorder. In the study participant (CMC 45) who met clinical diagnostic criteria for Aicardi syndrome (a condition without a known genetic cause), a negative genome sequencing result decreased the likelihood of a mendelian mimic. In a young child with neurological deficits associated with a perioperative event and otherwise putatively isolated transposition of the great arteries (CMC 28), a negative genome sequencing result similarly decreased the likelihood of a multisystem genetic syndrome. Such potential advantages of increasingly comprehensive genetic testing are deserving of further study.^73^

### Limitations

This study has some limitations. It was a single-center study, and the precise criteria for CMC enrollment in structured complex care programs differ by institution and region. The extensive phenotyping and availability of clinical ES may have been associated with the number and nature of the diagnoses made with genome sequencing in our cohort. A detailed comparison of phenotypic features between these study participants and the full CMC cohort from which they were ascertained was not possible. The diagnostic yield of genome sequencing in an unselected group of testing-naive CMC remains unknown. The systematic interpretation of many types of genomic variation (eg, complex structural variants) identified by genome sequencing remains challenging. The identification of pharmacogenetic variants was also beyond the scope of this initial study.^77^

In contrast to CMA and ES, genome sequencing is not yet widely available as a clinical test. This study was not designed to compare genome sequencing with ES. Proven advantages of genome sequencing germane to its use as a first-tier test include improved coverage of exonic regions as well as comprehensive detection of all sequence and structural variation in the nuclear and mitochondrial DNA.^8,18^ The added value of genome sequencing compared with ES is expected to increase over time as variant calling algorithms and annotation improve and as patient and control databases accumulate more genome sequencing data. As illustrated by the biallelic variants in *JAM3* in CMC 31 (eTable 3 in the Supplement), it remains challenging to classify novel deep intronic variants as likely pathogenic or pathogenic without dedicated functional studies that are beyond the scope of most clinical laboratories. At present, however, most diagnosed mendelian disorders are caused by exonic SNVs or large CNVs. If trio ES has already been performed on a clinical basis, reannotation and reanalysis of the existing data are likely a more cost-effective strategy than genome sequencing in the short term.^78,79,80^

## Conclusions

Children with medical complexity require interventions that differ in key ways from general care.^1,6^ Genome sequencing has the potential to increase the proportion of CMC for whom diagnoses are established. As a first-tier test, we speculate that genome sequencing could reduce the time and emotional burden of the diagnostic process and reduce health care system costs.^5^ Additional omic technologies,^81^ such as RNA sequencing^82,83,84^ and genome-wide DNA methylation testing,^85,86^ may further increase diagnostic yield in this population when used as an adjunct to genome sequencing. Beyond disease-specific therapeutics,^69,70^ having a confirmed molecular diagnosis will be a prerequisite to participating in gene therapy and genome editing trials. In time, we anticipate that genome sequencing will be a standard-of-care genetic test for undiagnosed CMC.

## Notes

Supplement. **eFigure 1.** Total Count of HPO Terms for the Proband Cohort (n = 49), by Phenotype Category **eFigure 2.** Total Number of Probands With at Least One HPO Term in a Phenotype Category **eFigure 3.** Total Count of Clinical Genetic Tests in Each Category Performed in the Proband Cohort (n = 49) **eFigure 4.** Total Number of Probands With at Least One Test in a Clinical Genetic Test Category **eFigure 5.** Mean Depth of Coverage of the Genome Sequencing Data **eFigure 6.** Median Count of Features by HPO Category in the Diagnosed and Undiagnosed Subgroups **eTable 1.** Individuals With Suspected or Partial Primary Diagnoses Known at the Time of Study Recruitment **eTable 2.** Demographic and Clinical Features in the Proband Study Cohort **eTable 3.** Selected Variants of Uncertain Diagnostic Significance Identified in the Proband Study Cohort
