# Supplementary material for: Genome Sequencing as a Diagnostic Test in Children With Unexplained Medical Complexity
Source: JAMA Netw Open. 2020 Sep 22;3(9):e2018109. doi: 10.1001/jamanetworkopen.2020.18109 (PMC7509619; doi:10.1001/jamanetworkopen.2020.18109)
Supplement: Supplement. — eFigure 1. Total Count of HPO Terms for the Proband Cohort (n = 49), by Phenotype Category eFigure 2. Total Number of Probands With at Least One HPO Term in a Phenotype Category eFigure 3. Total Count of Clinical Genetic Tests in Each Category Performed in the Proband Cohort (n = 49) eFigure 4. Total Number of Probands With at Least One Test in a Clinical Genetic Test Category eFigure 5. Mean Depth of Coverage of the Genome Sequencing Data eFigure 6. Median Count of Features by HPO Category in the Diagnosed and Undiagnosed Subgroups eTable 1. Individuals With Suspected or Partial Primary Diagnoses Known at the Time of Study Recruitment eTable 2. Demographic and Clinical Features in the Proband Study Cohort eTable 3. Selected Variants of Uncertain Diagnostic Significance Identified in the Proband Study Cohort [file jamanetwopen-e2018109-s001.pdf]

Supplementary Online Content

Costain G, Walker S, Marano M, et al. Genome sequencing as a diagnostic test in children with unexplained medical complexity. *JAMA Netw Open.* 2020;3(9):e2018109. doi:10.1001/jamanetworkopen.2020.18109

eFigure 1. Total Count of HPO Terms for the Proband Cohort (n=49), by Phenotype Category
eFigure 2. Total Number of Probands With at Least One HPO Term in a Phenotype Category
eFigure 3. Total Count of Clinical Genetic Tests in Each Category Performed in the Proband Cohort (n=49)
eFigure 4. Total Number of Probands With at Least One Test in a Clinical Genetic Test Category
eFigure 5. Mean Depth of Coverage of the Genome Sequencing Data
eFigure 6. Median Count of Features by HPO Category in the Diagnosed and Undiagnosed Subgroups
eTable 1. Individuals With Suspected or Partial Primary Diagnoses Known at the Time of Study Recruitment
eTable 2. Demographic and Clinical Features in the Proband Study Cohort
eTable 3. Selected Variants of Uncertain Diagnostic Significance Identified in the Proband Study Cohort

This supplementary material has been provided by the authors to give readers additional information about their work.

© 2020 Costain G et al. *JAMA Network Open.*

eFigure 1. Total Count of HPO Terms for the Proband Cohort (n=49), by Phenotype Category

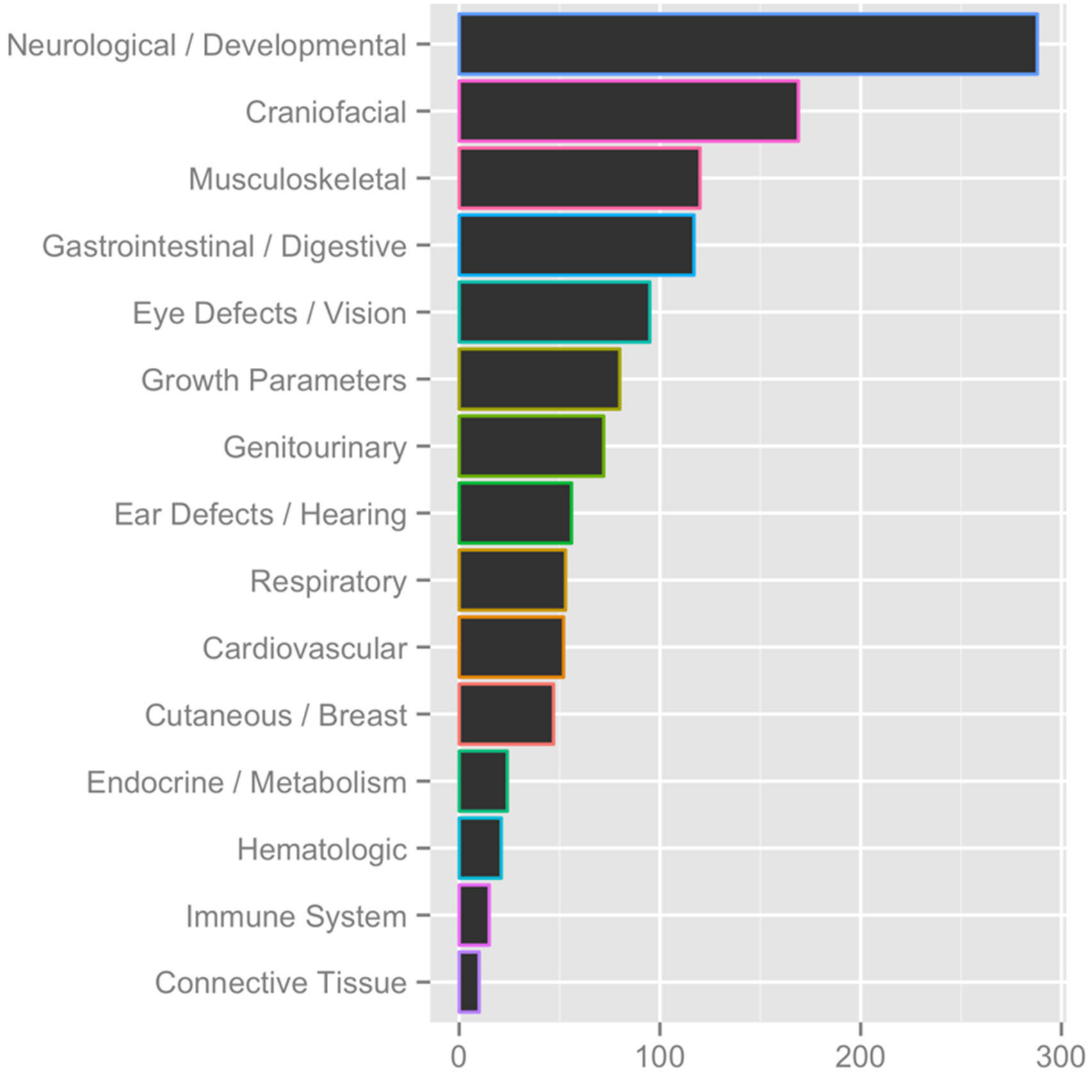

© 2020 Costain G et al. *JAMA Network Open.*

eFigure 2. Total Number of Probands With at Least One HPO Term in a Phenotype Category

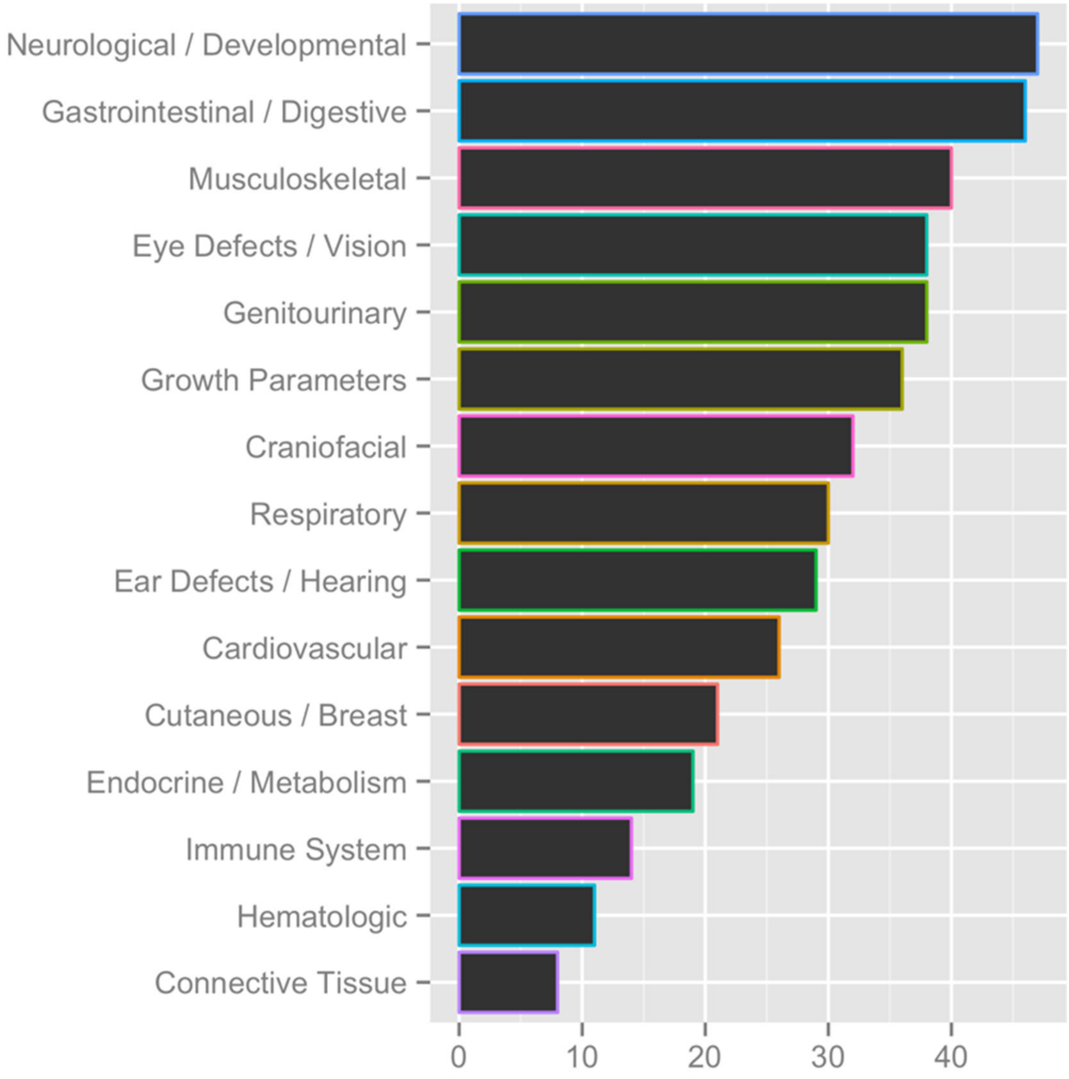

© 2020 Costain G et al. *JAMA Network Open.*

eFigure 3. Total Count of Clinical Genetic Tests in Each Category Performed in the Proband Cohort (n=49)

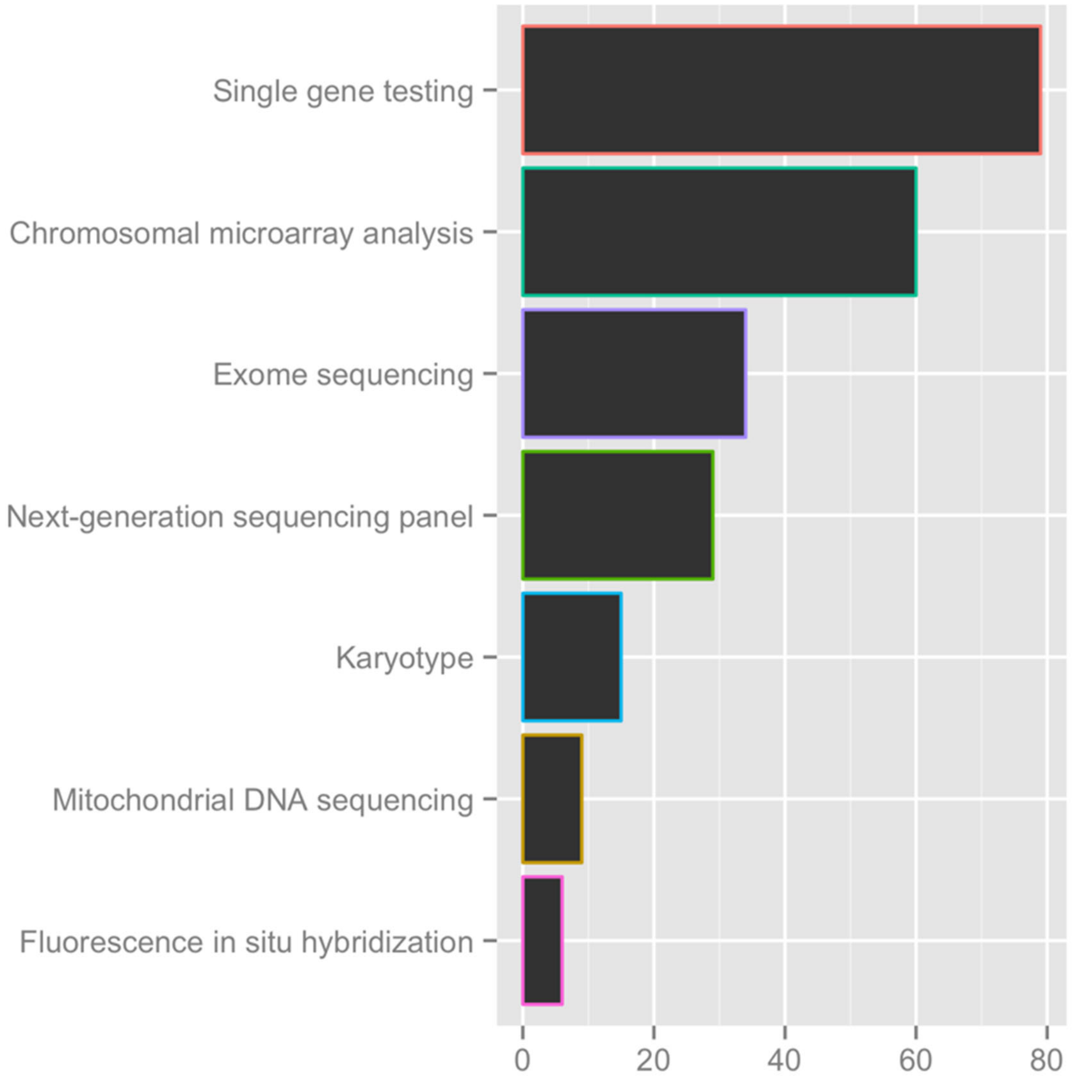

© 2020 Costain G et al. *JAMA Network Open.*

eFigure 4. Total Number of Probands With at Least One Test in a Clinical Genetic Test Category

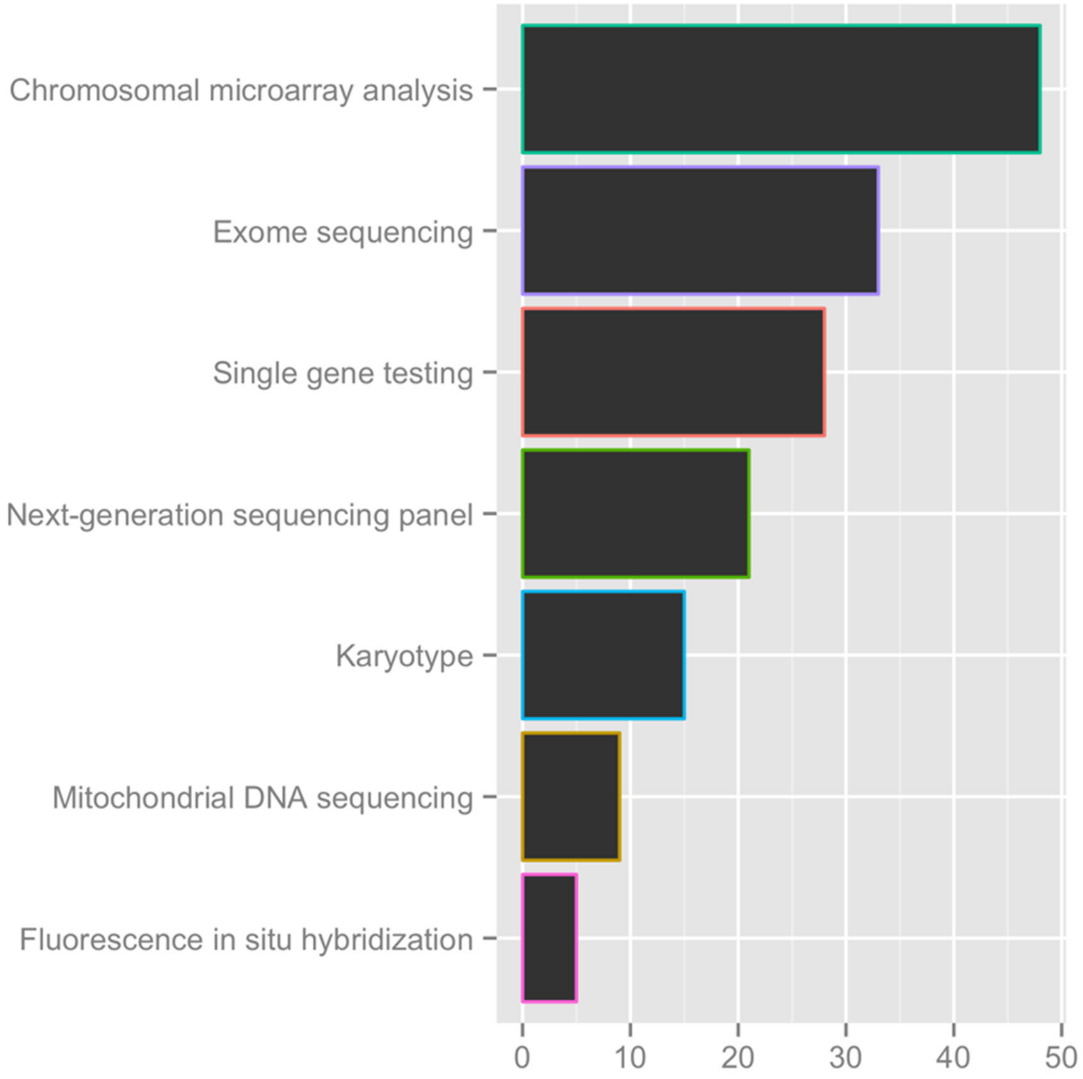

© 2020 Costain G et al. *JAMA Network Open.*

eFigure 5. Mean Depth of Coverage of the Genome Sequencing Data

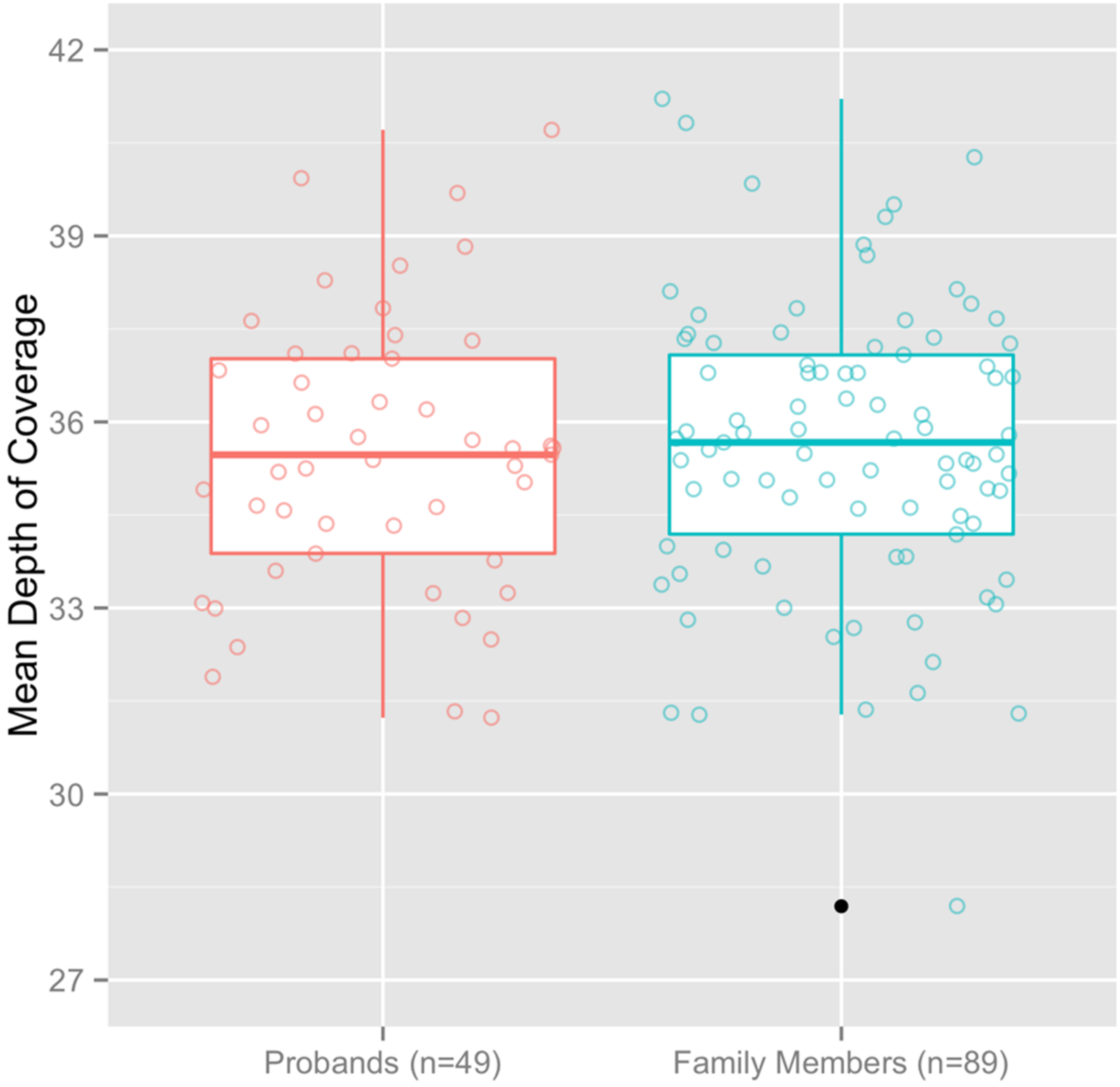

eFigure 6. Median Count of Features by HPO Category[a] in the Diagnosed and Undiagnosed Subgroups

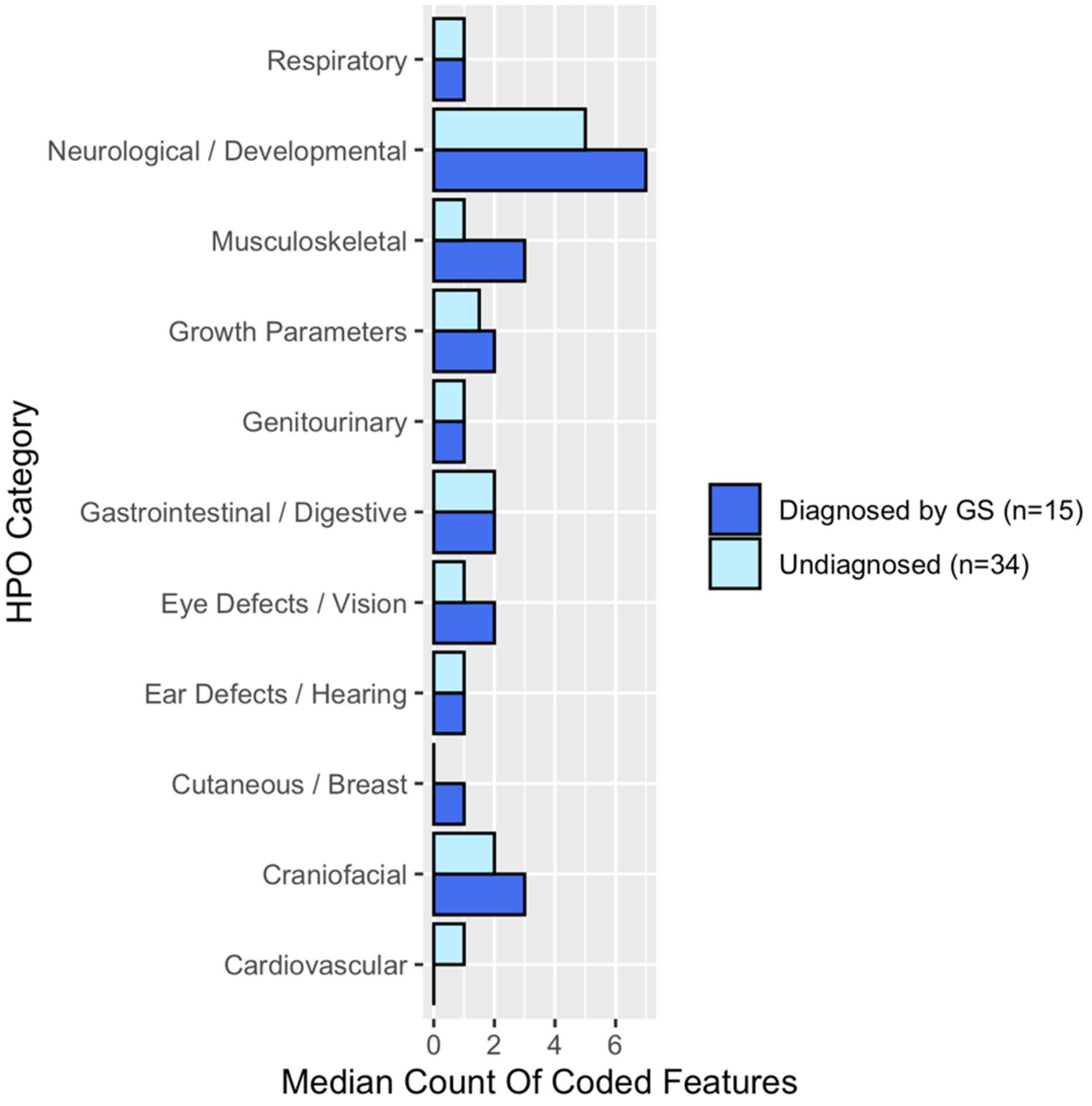

[a]The categories "Hematologic", "Immune System", "Connective Tissue", and "Endocrine / Metabolism" are not displayed because the median count was 0 in both subgroups.

© 2020 Costain G et al. *JAMA Network Open.*

eTable 1. Individuals With Suspected or Partial Primary Diagnoses Known at the Time of Study Recruitment

Study ID	***Sex***	***Gene***	***Condition (MIM # Phenotype)***	***IP***	***Variant Details (Zygosity) [Transcript]***	***Origin***	***Reasons for further genetic testing***
CMC 02	M	-	Down syndrome (190685)	-	47,XY,+21	dn	Severity of developmental delays, seizure disorder, and feeding difficulties
CMC 23	F	*HNF4A*	Fanconi renotubular syndrome 4, with maturity-onset diabetes of the young (616026)	AD	c.187C>T / p.(R63W) (het) [NM_175914]	pat	Multiple major congenital anomalies
CMC 32	M	*SOX5*	Lamb-Shaffer syndrome (616803)	AD	c.928T>A / p.(C310S) (het) (NM_006940)	dn	Type I transferrin isoform profile, and variant not classified as pathogenic
CMC 43	M	*KRAS*	Noonan syndrome 3 (609942)	AD	c.40G>A / p.(V14I) (het) [NM_004985]	dn	Treatment-resistant hyperkinetic movement disorder
CMC 46	M	*ATP6AP2*	Mental retardation, X-linked, syndromic, Hedera type (300423)	XL	c.301-11_301-10delTT / p.(?) (hem) [NM_005765]	dn	Inflammatory bowel disease, and variant not classified as pathogenic
CMC 49	M	*EDA*	Ectodermal dysplasia 1, hypohidrotic, X-linked (305100)	XL	c.458G>A / p.(R153H) (hem) [NM_001005612]	mat	Multiple major congenital anomalies

AD, autosomal dominant; dn, *de novo*; F, female; hem, hemizygous; het, heterozygous; IP, inheritance pattern; M, male; mat, maternal; MIM, Mendelian Inheritance in Man® (www.omim.org); pat, paternal; XL, X-linked

© 2020 Costain G et al. *JAMA Network Open.*

eTable 2. Demographic and Clinical Features in the Proband Study Cohort

	**Total (n=49)**		**Diagnosed by GS (n=15)**		**No diagnosis by GS (n=34)**		**p-value**[a]
	Number	(%)	Number	(%)	Number	(%)	
Male sex	29	(59.1)	7	(46.6)	22	(64.7)	ns
Clinical ES	33	(67.3)	9	(60.0)	24	(70.5)	ns
Positive family history[b]	6	(12.2)	3	(20.0)	3	(8.8)	ns
Parental consanguinity	5	(10.2)	2	(13.3)	3	(8.8)	ns
European/Caucasian ethnicity	26	(53.0)	8	(53.3)	18	(52.9)	ns
	Median	(range)	Median	(range)	Median	(range)	
Age in years	6.9	(0.6-17.8)	8	(4.5-17.8)	4.9	(0.6-13.2)	0.003
Coded HPO terms[c]	24	(6-58)	26	(11-45)	23	(6-58)	ns

ES, exome sequencing; GS, genome sequencing; HPO, Human Phenotype Ontology; ns, not significant (p>0.05)

[a]Fisher’s exact test for comparison of proportions, and Kruskal–Wallis test for comparison of medians.
[b]First-degree relative with at least partial phenotypic overlap
[c]See text for details.

© 2020 Costain G et al. *JAMA Network Open.*

eTable 3. Selected Variants of Uncertain Diagnostic Significance Identified in the Proband Study Cohort

Study ID	*Sex*	*Selected Features*	*Gene*	*Variant Details (Zygosity) [Transcript]*	*Origin*
Known disease genes					
CMC 01	F	Congenital microcephaly, GDD/ID, seizures	*MED23*	c.3957+5G>A (hom) [NM_015979]	mat/pat
CMC 03	F	SMMCI	*CDON*	c.2674G>A / p.(G892S) (het) [NM_016952]	mat[a]
CMC 04	M	GDD/ID, seizures, hypotonia, structural CNS anomalies	*PLCB1*	c.246+197G>A (hom) [NM_015192]	mat/pat
CMC 07	F	GDD, hypotonia, structural CNS anomalies	*KIF1A*	c.4566-66C>T (het) [NM_004321]	dn
CMC 28	M	GDD/ID, seizures, microcephaly, congenital anomalies	*THOC2*	c.623A>G / p.(N208S) (hem) [NM_001081550]	mat
CMC 29	M	GDD, congenital heart disease	*CTBP1*	c.107G>A / p.(R36Q) (het) [NM_001328][b]	dn
CMC 31	M	Intracranial hemorrhage, congenital cataracts	*JAM3*	c.410-1G>A (het) [NM_032801]	mat
				c.256+1260G>C (het) [NM_032801]	pat
CMC 37	M	Potter sequence, GDD	*POLA1*	c.3835T>G / p.(F1279V) (hem) [NM_016937]	mat
CMC 50	F	GDD, MCA	*TLK2*	c.532-1571_831+4del (het) [NM_006852][c]	uk[d]
Genes not yet associated with a Mendelian disorder					
CMC 04	M	GDD/ID, seizures, structural CNS anomalies	*MAGEE1*	c.877_984del / p.(293_328del) (hem) [NM_020932]	dn
CMC 18	F	GDD, MCA, nystagmus	*SF3B3*	c.2012A>G / p.(N671S) (het) [NM_012426]	dn
CMC 26	M	Severe achalasia	*ANKRD13B*	c.1761C>G / p.(D587E) (het) [NM_152345]	dn[e]
CMC 32	M	Seizures, GDD, hypotonia, sensorineural hearing loss	*DHRSX*	c.643C>T / p.(L215F) (het[f]) [NM_145177]	mat[e]

© 2020 Costain G et al. *JAMA Network Open.*

			DHRSY	c.541G>T / p.(V181F) (het[f]) [NM_145177]	pat[e]
CMC 33	M	Arthrogryposis, hypotonia	*CAPRIN1*	c.1338A>T / p.(Q446H) (het) [NM_005898]	dn
CMC 34	M	Neurodegenerative disorder, aplastic anemia	*KLHL12*	c.1700A>T / p.(D567V) (het) [NM_001303051]	dn
CMC 41	M	Congenital right cerebral hypoplasia, GDD/ID	*PLXNC1*	c.1400C>A / p.(A467D) (het) [NM_005761]	dn

CNS, central nervous system; dn, *de novo*; F, female; GDD, global developmental delay; hem, hemizygous; het, heterozygous; hom, homozygous; ID, intellectual disability; M, male; mat, maternal; MCA, multiple congenital anomalies; pat, paternal; SMMCI, solitary median maxillary central incisor syndrome; uk, unknown

[a]Mother does not have features of SMMCI on exam. We questioned whether there could be a gene–environment interaction with another risk factor present in this family (maternal type 1 diabetes mellitus).
[b]ClinVar Accession Number: VCV000426724.2 (same patient).
[c]chr17:60628092_60637491del
[d]Not inherited from mother.
[e]Variant also present in similarly affected brother.
[f]Gene is within the pseudoautosomal region.

© 2020 Costain G et al. *JAMA Network Open.*
